# Evaluating the Immunogenicity and Safety of a Smallpox Vaccine to Monkeypox in Healthy Japanese Adults: A Single-Arm Study

**DOI:** 10.3390/life13030787

**Published:** 2023-03-14

**Authors:** Noriko Tomita, Eriko Morino, Junko Terada-Hirashima, Yukari Uemura, Yosuke Shimizu, Sho Saito, Tetsuya Suzuki, Nobumasa Okumura, Haruka Iwasaki, Hideki Ebihara, Masayuki Shimojima, Wataru Sugiura, Norio Ohmagari, Mugen Ujiie

**Affiliations:** 1 Center for Clinical Sciences, National Center for Global Health and Medicine, Tokyo 162-8655, Japan; 2 Disease Control and Prevention Center, National Center for Global Health and Medicine, Tokyo 162-8655, Japan; 3 National Institute of Infectious Disease, Tokyo 162-8640, Japan

**Keywords:** mpox, smallpox vaccine, single-arm open-label study, immunogenicity against mpox, safety of smallpox vaccine

## Abstract

Monkeypox (mpox) is an acute exanthematous disease caused by the monkeypox virus (MPXV). Since May 2022, patients with mpox have been reported worldwide, mainly in Europe and the Americas. In Japan, LC16”KMB,” which is a smallpox vaccine derived from a dried cell culture, against mpox, has been approved. Although inoculation with a smallpox vaccine has been recommended to prevent MPXV infection, the immunogenicity of the smallpox vaccine against the MPXV is unclear, and information regarding postvaccination safety is scarce. We present the protocol for a single-arm open-label study to investigate the immunogenicity and safety of LC16”KMB” against the MPXV in healthy Japanese adults. The primary endpoint is the seroconversion rate of neutralizing antibodies against the MPXV on postvaccination day 28. The secondary endpoints are the seroconversion rates against the MPXV on postvaccination days 14 and 168; the seroconversion rates against the vaccinia virus on postvaccination days 14, 28, and 168; the incidence of mpox until day 168; and adverse and serious adverse events until postvaccination days 28 and 168. These results will pave the way for larger comparative studies using other smallpox vaccines to evaluate the test vaccine’s safety and efficacy in preventing mpox.

## 1. Introduction

Monkeypox (mpox) is a viral zoonosis that is caused by monkeypox viruses (MPXVs), which belong to the poxvirus family. Their natural hosts have been rodents, thereby forming a cycle of infection between rodents and monkeys in rainforests extending from Central Africa to West Africa. The first case of human infection was confirmed in the Democratic Republic of the Congo in 1970. Human infections are reportedly caused by virus-infected monkey and rodent bites, as well as the consumption of undercooked meat. In addition, the spread of the infection from person to person has been reported to occur through routes such as droplets and interpersonal contact [1,2,3,4]. Although outbreaks have persisted mainly from Central to Western Africa [5], sporadic outbreaks have been reported in individuals returning from endemic areas outside Africa [6]. In 2003, an outbreak reported in Texas, USA, was probably caused by imported rodents. Mpox usually develops after an incubation period of 6–13 (up to 5–21) days after exposure to the virus [7]. After the incubation period, symptoms such as fever, headache, lymphadenopathy, and muscular involvement persist for about 1–5 days, followed by a rash. Efflorescence appears mostly on the face and extremities, and gradually elevates, accompanied by bullae, pustules, and crusts, which heal 2–4 weeks after the onset [6,8]. The case fatality rate is reported to be 0–11% [9], and it tends to be higher, especially in children [10] and immunocompromised patients who are susceptible to serious illness [11]. In May 2022, patients with mpox without a history of overseas travel were reported in the UK, and subsequent cases were reported in Europe and the United States. In July 2022, the World Health Organization (WHO) declared mpox a “public health emergency of international concern.” Since July 2022 through November, seven mpox cases have been confirmed in Japan. Considering the epidemic situation, the number of infected persons in Japan may increase in the future; therefore, taking measures to prevent the spread of the infection is necessary. As of 27 December 2022, a total of 83,497 mpox cases were confirmed, and 72 deaths had been reported to the WHO from 110 countries [12].

Currently, curative drugs for mpox include antivirals such as tecovirimat, brincidofovir, and cidofovir. Tecovirimat, developed and manufactured by SIGA Technologies, was approved for mpox and other orthopoxvirus infections in the European Union, England, Scotland, and Wales [13,14]. Tecovirimat can be used for the treatment of human non-variola orthopoxvirus infection under an expanded access IND protocol [15], a clinical trial protocol (ClinicalTrials.gov Identifier: NCT05534984) in the United States, and a specific clinical study in Japan (Clinical Trial Plan Number: jRCTs031220169; https://jrct.niph.go.jp/latest-detail/jRCTs031220169; registration date: 28 June 2022). Brincidofovir and cidofovir have also been used for mpox in small number of cases [16]. Brincidofovir is available under FDA-authorized single-patient emergency used IND [13]. In Japan, there is no approved therapeutic drug, and vaccination is implemented as prophylaxis. The smallpox guideline (fifth edition) suggests that there may be cross-immunity between the smallpox virus, vaccinia virus, and MPXV [17]. In fact, the smallpox vaccine has shown to be effective against mpox [10,18]. Moreover, according to a report by the Centers for Disease Control and Prevention on 5 December 2022 [19], vaccination within 4 days after exposure to the MPXV is effective in preventing mpox infection, and vaccination 4–14 days after exposure is still beneficial against the infection.

The dried cell-cultured smallpox vaccine LC16”KMB” (LC16), which is a smallpox vaccine developed in Japan and manufactured by KM Biologics Co. Ltd., is an attenuated live vaccine against smallpox derived from the LC16m8 strain. The LC16 was prepared using the Lister strain, which was used as the parent strain during the eradication phase of smallpox. In August 2022, an additional indication for the LC16 was approved for the prevention of mpox onset in Japan.

Several reports have demonstrated the efficacy of the LC16 in preventing the development of mpox. A randomized, double-blind, controlled trial was conducted in the United States between 2004 and 2005 to investigate the safety of LC16 and neutralizing antibodies against poxviruses through comparisons with a first-generation vaccine, Dryvax (Wyeth Laboratories, Inc., Marietta, PA, USA) [20]. All 125 subjects vaccinated with the LC16 developed local skin reactions at the injection sites (“take”), with 26 randomly selected subjects developing neutralizing antibodies against the MPXV and other vaccinia virus strains (NYCBH strains, Lister strains, and LC16 m8 strains). Other epidemiological data from mpox in the Republic of Zaire over a five-year period (1980–1984) show that smallpox vaccination prevents the development of mpox by approximately 85% [18].

Meanwhile, several studies have demonstrated the effectiveness of the MVA-BN vaccine (manufactured by Bavarian Nordic A/S), a third-generation smallpox vaccine similar to the LC16, in mpox prevention. The MVA-BN was approved in the United States for the prevention of mpox in 2019, and the WHO has recommended the use of the MVA-BN and LC16 for the prevention of mpox [21]. Hazra et al. reported mpox infection after a single dose of the MVA-BN in 400 patients who tested positive for mpox. Ninety patients tested positive for mpox at least 1 day after receiving a single dose of the MVA-BN, and 69 mpox cases (77%) occurred within 14 days of the vaccination. Meanwhile, eight mpox cases occurred 28 days after the vaccination. These results indicated that mpox infections mainly occurred within 14 days after a single dose of the MVA-BN, implying that complete effectiveness had not yet been achieved [22]. A study conducted in 32 United States jurisdictions comparing the incidence of mpox among unvaccinated men and those who received at least one dose of the MVA-BN vaccine among 5402 men aged 18–49 years [23] revealed that the mean incidence of mpox among unvaccinated men was 14.3 times higher (95% CI: 5.0–41.0) than that among those who received one dose of the MVA-BN vaccine more than 14 days ago. This suggests that a single dose of the MVA-BN vaccine offers some protection against mpox infection. In a study to determine whether childhood smallpox vaccination altered the clinical presentation of the MPXV infection, 298 mpox-infected patients were followed up for at least 7 days [24]. As a result, acute systemic symptoms such as fever and somatic pain were less frequent in the vaccinated group, whereas local skin disease without systemic symptoms (defined as 0–3 lesions) was more frequent in the vaccinated group, with an odds ratio of 4.17 in the vaccinated group (95% CI: 1.63–10.65, p = 0.003). In addition, several studies have reported the safety of the LC16 vaccination. In 1974, the LC16 was administered to approximately 50,000 children in Japan, and clinical manifestations were observed in approximately 10,000 of them. The percentage of “take,” or successful vaccination, was 95.1%, with fever (4–14 days postvaccination) occurring in 7.7% of cases. Adverse reactions, such as vaccinal local skin lesions in 28 cases, autoinoculation in 9 cases, allergic eczema in 8 cases, and febrile convulsions in 3 cases, were observed [25]. Then, the phase IV postmarketing surveillance of the LC16 was conducted in 268 adults between 2005 and 2010 in Japan. The ”take” was observed in 185 of 196 (94.4%) first-time recipients, and 58 of 71 (81.7%) re-recipients of the LC16. Adverse events were identified in 27.0% of first-time recipients and 5.6% of re-recipients. Major adverse events included axillary lymphadenopathy in 52 (19.4%) patients, and inoculation site erythema in 14 (5.2%) patients [26]. Moreover, a clinical study was conducted to evaluate the safety of LC16 in healthy men and women aged 18–55 years, between 2002 and 2005 [27]. In this study, 3221 patients were vaccinated. Beneficial responses were confirmed in 1443/1529 (94.4%) first-time recipients, and in 1465/1692 (86.6%) re-recipients. No serious adverse events were reported 10–14 days after vaccination; however, two severe cases occurred during the 30-day postvaccination interval. One patient developed allergic dermatitis, whereas the other developed erythema multiforme. The causal relationship with the vaccine could not be denied in both cases.

Thus, several studies to investigate the safety of the LC16 were conducted by 2010; however, routine vaccination against smallpox has not been conducted since 1976 in Japan because of the eradication of smallpox. Consequently, the latest information regarding the safety of smallpox vaccination has not been accumulated.

Based on the preceding discussion of the efficacy and safety of smallpox vaccines against the MPXV, this clinical trial is designed to confirm the immunogenicity and occurrence of adverse events after the inoculation of the LC16 vaccine, which can be manufactured and made readily available in Japan. The results of the study can be used to estimate the efficacy of the vaccine in preventing mpox, and provide detailed information on the safety of this vaccine in healthy Japanese adults. This trial was registered with the Japan Registry of Clinical Trials (Clinical Trial Plan Number: jRCTs031220171; https://jrct.niph.go.jp/latest-detail/jRCTs031220171; first registration date: 30 June 2022).

## 2. Methods

### 2.1. Information on the Smallpox Vaccine (Test Vaccine) Used in Clinical Research

(1) Name of the test vaccine (generic and brand names)

KMBs, freeze-dried cell culture smallpox vaccine LC16.

(2) Vaccination

Fifty subjects employed at the National Center for Global Health and Medicine who provide medical care to patients with mpox will be enrolled. Dissolve the test vaccine in 0.5 mL of the attached solvent (water for injection with 20 vol% glycerin), dip the designated bifurcated needle in the vaccine diluent, and puncture using the bifurcated needle 5 times for the first vaccination and 10 times for subsequent vaccinations.

### 2.2. Purpose of the Clinical Research

This study aimed to confirm the immunogenicity and safety of vaccinating healthy Japanese adults with the smallpox vaccine against mpox.

### 2.3. Content of the Clinical Research

(1) Types and methods of clinical trials

A single-arm, open-label study was to be conducted at a single center, the National Center for Global Health and Medicine (NCGM).

(2) Inclusion and exclusion criteria for the subjects of the clinical research

Trial criteria and the rationale for their setting are shown in Table 1.

In addition, if the vaccinated person falls into any of the following categories, the vaccine should be carefully administered through a medical examination and the judgment of the adequacy of the vaccination, taking into account the individual’s health condition and the vaccine’s composition.

- Subjects with underlying diseases such as shock or anaphylaxis (hives, dyspnea, lip edema, pharyngeal edema, etc.) to gelatin-containing preparations or foods containing gelatin;
- Subjects with underlying diseases, such as cardiovascular disease, kidney disease, liver disease, hematologic disease, or developmental disorder;
- Subjects who have experienced a fever within 2 days after vaccination, or who have had symptoms suspicious of allergy, such as a systemic rash (not applicable if it is confirmed that the causative ingredient is not included in the test vaccine);
- Subjects with a history of convulsions;
- Individuals with a previous diagnosis of immunodeficiency, and relatives with congenital immunodeficiency;
- Subjects who may be allergic to the ingredients of the test vaccine.

(3) Criteria for discontinuation
   Studies will be discontinued for participants who meet the following criteria for discontinuation:
  ① When research subjects request to withdraw consent,
  ② When compliance with the study protocol becomes impossible,
  ③ When the entire study is discontinued,
  ④ Difficulty in continuing the study as per the judgment of the investigator or subinvestigator.
(4) Treatment and observation periods

The vaccine will be administered as a single dose. Blood tests will be performed before vaccination and at 14, 28, and 168 days after vaccination, and the subject will be followed up until 168 days.

### 2.4. Study Schedules

The smallpox vaccine will be administered after obtaining written informed consent according to the timeline shown in Table 2 (Visit 1). Each subject’s body temperature will be measured daily until day 14 after the vaccination, and symptoms and adverse events will be recorded in the subject’s diary.

Blood sampling will be performed on days 14, 28, and 168 postvaccination, and the investigator, subinvestigator, or clinical research coordinator will check the presence, severity, and outcomes of the adverse events (visits 2, 3, and 4).

In the event of contact with a patient with mpox, the date and time of contact and the status of contact will be recorded. When mpox occurs, the date of onset shall be recorded. If immunosuppressive therapy is initiated during the procedure, information on the reason for the therapy and treatment details will be collected.

The medical interview form will be used, when appropriate, to collect information on adverse events, history of contact with patients with mpox, concomitant medications, etc.

(1) Subjects’ characteristics
  ① Sociodemographic characteristics of the research subjects (age, sex, occupation, race, etc.),
  ② Professional exposure (e.g., working contact with patients with smallpox and mpox),
  ③ Information on diseases and drugs that may affect either antibody production or the Th1/Th2 ratio (as appropriate),
  ④ Medical examination information (medical history, etc.) as needed,
  ⑤ Adverse reaction information (fever, malaise, etc., according to the subject’s diary),
  ⑥ Content of the vaccination questionnaire,
  ⑦ Presence or absence of a history of smallpox vaccination.
(2) Investigation items for immunogenicity endpoints
  ① Timing of blood sampling: visit 1 (before the test vaccine inoculation), visit 2 (day 14 postvaccination), visit 3 (day 28 postvaccination), and visit 4 (day 168 postvaccination). However, visits 2 and 3 should be allowed for +4 days, and visit 4 for ±7 days.
  ② Measurements:
    (a) Neutralizing antibodies (days 0, 14, 28, and 168) against the MPXV,
    (b) Neutralizing antibodies (days 0, 14, 28, and 168) against the vaccinia virus (inoculated virus) measured at the National Institute of Infectious Diseases (NIID) using serum and plasma,
    (c) The presence or absence of the “take”.
     At 10–14 days postvaccination, the vaccine shall be clearly inoculated to indicate that immunity has been acquired (a condition in which local inflammatory reactions, such as redness, swelling, warmth, induration, and blisters at the inoculation site can be confirmed).
  ③ Blood collection volume: 21 mL per visit (5 mL for the sera × 1, 8 mL for the plasma (anticoagulant and sodium citrate) × 1, and 8 mL for the plasma (anticoagulant and heparin sodium) × 1).
  ④ Processing method: Five milliliters of blood serum samples will be collected using blood collection tubes containing serum separators. After blood collection, samples should be kept at room temperature and transported to the NIID within 2 h. At the NIID, after confirming coagulation, the serum is centrifuged (3000 rpm for 10 min) and isolated. The obtained serum should be kept frozen at −20 °C or lower temperatures. The serum is used to measure the number of MPXV-neutralizing antibodies and vaccinia virus-neutralizing antibodies. The date and time of specimen collection should be recorded in the source documents. For blood plasma, 8 mL × 1 blood samples should be collected using a blood collection tube for CPT^TM^ (BD, Franklin Lakes, NJ, USA) mononuclear cell isolation (sodium citrate) and a blood collection tube for CPT^TM^ mononuclear cell isolation (sodium heparin). The sample will be transported to the NIID within 2 h of blood sampling. At the NIID, centrifugation will be performed immediately (3000 rpm for 20–30 min). Plasma and peripheral blood mononuclear cells shall be collected from the blood collection tubes after centrifugation, and cryopreserved at −80 °C or lower temperatures.
     The plasma will be used for the measurement of the MPXV (MPXV_Zr599: Congo Basin strain; MPXV_Liberia: West African strain) or vaccinia virus-neutralizing antibodies. The date and time of specimen collection will be recorded in the source documents. Peripheral blood mononuclear cells will be cryopreserved only for subjects who have consented to the storage and secondary utilization of the remaining samples.
  ⑤ Labeling and transportation methods of blood collection tubes and sample storage containers: Labels will contain the test subject name (abbreviation), subject identification code, blood sampling time, and blood sampling date, and will be attached to the blood collection tubes and sample storage containers. Specimens will be placed in dedicated sample transport boxes and transported to the NIID.
(3) Evaluation and documentation of adverse events
   The investigator shall record the following items on the Case Report Form in relation to adverse events that occur in the research subjects during visits 1–4.
  ① Name of the adverse event,
  ② Incidence date of the adverse event,
  ③ Severity (see severity classification of adverse events),
  ④ Seriousness (see the reporting of diseases to the NCGM-certified Clinical Research Review Board (research using unapproved or off-label drugs, etc.)),
  ⑤ Presence and content of treatment,
  ⑥ Outcomes (recovery including remission, recovery but with sequelae, not recovered, death, or unknown) and date of outcomes,
  ⑦ Causal relationship with the test vaccine.
(4) Others
  ① If the subjects make contact with a patient with mpox or smallpox by visit 4 after study initiation, the timing and contact status should be recorded.
  ② If mpox is detected on visit 4 after study initiation, the date of diagnosis should be recorded.
  ③ Diseases involving immunity, and drugs initiated on visit 4 after study initiation should be recorded.
(5) Acceptable therapies (including emergency care) before and during the clinical research
  In the event of an adverse reaction associated with vaccination, the concomitant use of drugs required for symptomatic treatment is acceptable.
(6) Therapies prohibited before and during the clinical trial
  Vaccination other than the test vaccine is prohibited until visit 3.

### 2.5. Evaluation Items

(1) Primary endpoint

The neutralizing seroconversion is determined using the neutralizing antibody titer at visit 1 for each subject shown in Table 3.

In principle, clinical trials evaluating the efficacy of the smallpox vaccine against the MPXV should be conducted to evaluate its efficacy in preventing mpox. However, a long study period is required to assess the onset-preventive effect, which is not realistic, considering that this study is exploratory. To determine whether a larger validation study should be conducted in the future, neutralizing antibodies in the blood will be evaluated as an important item. In addition, the primary assessment will be conducted on day 28, i.e., the time confirming the common seroconversion of the neutralizing antibodies by the test vaccine.

(2) Secondary and exploratory endpoints

The details of secondary and exploratory endpoints are shown in Table 4.

(3) Procedure for measuring neutralizing antibody titers in sera following smallpox vaccination
  ① Neutralizing antibody response and the inoculation of cells:
    (a) Inactivate the human serum to be tested for neutralizing antibody titers at 56 °C for 30 min, and store it at 4 °C. In this study, three viruses (one vaccinia virus and two MPXVs: MPXV_Zr599 and MPXV_Liberia) will be used. Mix the disseminated cells with the serum–virus mixture on the same day.
    (b) Maintain RK13 cells in Dulbecco′s Modified Eagle′s Medium (DMEM) supplemented with 5% fetal bovine serum (FBS) and antibiotics (Pen Strep) at 37 °C in a humidified atmosphere of 5% CO_2_ in the air, with a passage approximately twice a week. Prepare an RK13 cell culture suspension and stir the cells at a concentration of 1 × 10^5^ cells/well in a cell culture plate. Shake the plate moderately to prevent bias in the cell suspension. Place the plate in a carbon dioxide incubator at a CO_2_ concentration of 5% at 37 °C for 1 day. Prepare three plates (one plate for each virus type) per serum specimen and three plates for standard serum.
    (c) Dilute the test serum 2-, 4-, 8-, 16-, 32-, 64-, and 128-fold using DMEM supplemented with 2% FBS and antibiotics (cell maintenance medium) in 96-well round-bottom plates. Because the test serum will be mixed with the same number of viruses, dilute it 4-, 8-, 16-, 32-, 64-, 128-, and 256-fold after mixing. Prepare 50 µL of each dilution in three wells for each virus.
    (d) Dilute the three viruses in the cell maintenance medium as follows:
      ▪ Vaccinia virus LC16m8: 2900-fold,
      ▪ MPXV Zr-599 strain: 660-fold,
      ▪ MPXV Liberia strain: 450-fold.
    (e) Using three rows of dilution columns prepared in (a)–(c) for each virus, add the same amount of virus prepared in (d) to each well, and allow it to stand for 1 day in the 5% carbon dioxide incubator at 37 °C.
    (f) After 18 h, remove the culture medium from the plates prepared in (b), and add 0.3 mL of fresh cell-maintaining medium per well. Add 100 µL of the serum-virus mixture prepared in (e) to the plate, shake moderately, and incubate in the 5% carbon dioxide incubator at 37 °C. After incubation for 72–96 h, remove the culture solution from the wells, add approximately 1 mL of 10% neutral buffered formalin per well, and let it stand at room temperature for at least 1 h (up to 3 days).
  ② Staining of cells with a crystal violet solution (0.1% crystal violet with 10% ethanol):
     Wash each well with tap water to remove formalin. Then, place a suitable volume of crystal violet solution in each well and let it stand at room temperature for 5 min. Remove the crystal violet solution from each well and wash with tap water.
  ③ Calculation of neutralizing antibody titers:
    (a) Compute the plaque count using the CTL Switchboard. For wells suspected of not being correctly counted, or for wells in which the number of plaques is severely reduced due to neutralization and thus not counted using the CTL Switchboard, two examiners should visually count the plaques to determine the agreed-upon number of plaques. In such cases, record the date of the analysis and the examiner’s name.
    (b) Determine the mean number of plaques in the test and standard serum-free specimens in each plate, and use 50% of this value as the threshold.
    (c) Starting with the wells with a low serum dilution ratio, determine the maximum serum dilution rate (half of the serum dilution rate at which the number of plaques is the same as or above the threshold for the first time) at which the number of plaques falls below the threshold in each dilution column. Subsequently, designate the geometric mean as the neutralizing antibody titer of the serum. If the maximum dilution rate of the test serum also shows that the number of plaques is below the threshold, change the dilution of the test serum to another two-fold serial dilution and perform retesting.
    (d) For a dilution column in which the number of plaques does not fall below the threshold, even when the dilution of the test serum is 4096-fold, the number of plaques is considered to be the maximum dilution factor at which the number of plaques falls below the threshold value of 4096-fold. However, if the number of plaques is not below the threshold of 4096-fold in all three dilution columns, the calculated neutralizing antibody titer is considered to be at least 4096-fold.
    (e) In two valid tests (e.g., Study A and Study B) conducted with dilution rates considered appropriate for the same test serum, the highest dilution rate used in Study A is given in the dilution column in Study A, and half of the highest dilution rate used in Study B is given in the dilution column in Study B; moreover, neutralizing antibody titers are calculated using a total of six dilutions obtained from each of the two tests if the following conditions are met:
      ▪ If there is a dilution column in which the number of plaques is below the threshold, even at the highest dilution rate in Study A;
      ▪ If there is a dilution column in which the number of plaques does not fall below the threshold, even at the lowest dilution rate in Study B.
  ④ Discontinuation of the test:

If it is determined that it is impossible to obtain a valid test result from the test before its completion, the test may be discontinued at that time. In such cases, the specimen will be withdrawn from the test, and the specimen ID and the reason for withdrawal will be recorded. In principle, retesting should start after the initial test results are established. However, if retesting is initiated before the initial test is completed, the initial test will be completed before retesting is completed. In addition, if the initial test is established and valid test results are obtained, the test shall be terminated without obtaining the results of the retest at that time. After specifying the ID of the target sample on the working record form, record it as “termination of the retest for confirming the results of the initial test.”

### 2.6. Statistical Analysis

(1) Analysis sets
  ① Full analysis set (FAS)
     The study population excludes ineligible subjects, unvaccinated subjects, and subjects who do not have data on the efficacy endpoints after inoculation.
  ② Safety set
    The study population, excluding unvaccinated subjects, will be included.
(2) Target sample size and rationale

The target sample size is 50 subjects.

Rationale: There are no previous data on neutralizing antibodies against the MPXV, and no previous data have evaluated the relationship between mpox prophylaxis and neutralizing antibody titers in Japanese individuals who received the smallpox vaccine, making it difficult to appropriately establish a threshold for neutralizing antibody titers that can be considered as effective against this virus. In this study, we aimed to confirm whether cross-immunity to the MPXV can be induced via vaccination, using this vaccine approved as a smallpox vaccine by evaluating the neutralizing antibody seroconversion rate against the MPXV. We set 50 as the number of cases for which rapid case registration and the analysis of specimens could be feasible.

If 45 of the 50 subjects (with some data not being available) were included in the analysis set, the probability that the lower limit of the 95% CI of the estimate would exceed 50% under the expectation that the neutralizing antibody seroconversion rate, which will be 75%, would exceed 90%. Studies on neutralizing antibody seroconversion rates against the vaccinia virus for this vaccine have reported 90.2% (95% CI: 81.2–99.3%) in cases without a history of vaccination, and 60.0% (95% CI: 52.3–67.7%) in cases with a history of vaccination. The above 75% is the antibody conversion rate that is assumed if a similar neutralizing antibody seroconversion rate was observed for the MPXV and if individuals with the same rate of cases with/without a history of vaccination were enrolled in the trial.

(3) Criteria for the discontinuation of clinical research
   When the investigator discontinues the study itself, he/she promptly reports the details of the study and its reasons to the NCGM Clinical Research Review Board (NCGM CRB) and the Ministry of Health, Labour, and Welfare (MHLW). Appropriate measures should be taken for the clinical research subject. The opinions of the NCGM CRB should be listened to regarding the timing of research completion, and the methods associated with the measurements of subjects, as needed. In addition, even if a notification of discontinuation is submitted, reports of disease and periodic reports shall be made until the completion of the clinical study. Discontinuation refers to the early termination of the study for any of the following reasons. In addition, interruption refers to the temporary suspension of case registration when the following reasons are suspected:
  ① When the safety of the study is deemed to be problematic, based on the information made available during this study;
  ② When the safety of this study is deemed to be problematic, based on the information from sources other than from this study;
  ③ When the significance of this study is denied, based on information from sources other than this test;
  ④ If the trial is judged as being difficult to complete, the follow-up and analysis periods, if discontinued, will be in accordance with the protocol, starting from the date of the last enrollment.
(4) Statistical analysis items and the analysis plan
   In all cases, analyses should be performed after the administration of the test vaccine has been completed and the data have been fixed. For all efficacy assessments, the primary analysis will be for FAS. Safety analyses will be conducted in the safety population. Details of the statistical analysis will be specified in the statistical analysis plan prepared separately before data fixation.
  ① Analysis of the backgrounds of the subjects
    Background factors will be tabulated using the safety analysis set and FAS.
  ② Primary endpoint analysis
     The following analyses will be performed using FAS as the analysis set:
     The proportion and 95% CI of subjects with the seroconversion of neutralizing antibodies to the MPXV on day 28 postvaccination will be calculated using serum specimens. Subgroup analyses according to age group, sex, race, previous smallpox vaccination history, diseases related to immunity and concomitant drugs, the occurrence of mpox, vaccinating physician, and the presence/absence of the ”take”, will be performed as well.
  ③ Secondary variable analysis
    ▪ Proportion of subjects with seroconverted neutralizing antibodies to the MPXV, and their 95% CIs in FAS on days 14 and 168 postvaccination. Additionally, the proportion and 95% CI of subjects with the seroconversion of neutralizing antibodies to the MPXV on day 28 postvaccination, using plasma specimen (anticoagulant and sodium citrate) and plasma specimen (anticoagulant and sodium heparin);
    ▪ Proportion of subjects with the seroconversion of neutralizing antibodies to the vaccinia virus in FAS on days 14, 28, and 168 postvaccination, with 95% CIs;
    ▪ Trends in neutralizing antibodies in cases of mpox contact during the observation period;
    ▪ Number of subjects with mpox, the proportions, and their 95% CIs;
    ▪ Number of subjects with the “take”, the proportions, and their 95% CIs;
  ④ Exploratory endpoints analysis
    ▪ Neutralizing antibody titers against the MPXV on days 14, 28, and 168, postvaccination;
    ▪ Neutralizing antibody titers against the vaccinia virus on days 14, 28, and 168, postvaccination.
  ⑤ Safety endpoint analysis
    The following analyses will be conducted in the safety analysis set.
    ▪ For all adverse events occurring between postvaccination and day 28, the number and percentage of subjects will be calculated for the entire adverse event and for each adverse event. In addition, deaths, nonfatal serious adverse events, and more severe (grade ≥ 3) adverse events will be listed.
    ▪ The number and proportion of subjects with the specified local adverse events, specified systemic adverse events, and unspecified adverse events, which are physician-reported outcomes, will be summarized for each type of adverse event.
    ▪ Descriptive statistics will be calculated for the maximum temperature from the moment of vaccination to 14 days postvaccination.
    ▪ Subject-reported outcomes will be tabulated according to adverse events.
  ⑥ Procedures for changing the original analysis plan

If there are changes to the original statistical analysis plan, the study plan or the statistical analysis plan should be revised and explained in the clinical research report.

### 2.7. Adverse Events, Diseases, etc.

(1) Procedures for collecting, recording, and reporting information on diseases, etc.

The investigator or subinvestigator will document all of the adverse events occurring during the first 28 days postvaccination, and serious adverse events occurring between day 29 and day 168 postvaccination, to determine a causal relationship between these adverse events and the clinical study. In NCGM, if serious adverse events occur, regardless of whether they are related to the test vaccine, they should be promptly reported to the administrators of the participating medical organizations, the NCGM Clinical Research Safety Management Office, and then to the NCGM CRB, as soon as they are aware of the serious adverse events.

(2) Reporting of diseases suspected to be attributable to the conduct of clinical research, etc., to the NCGM CRB (research using unapproved or off-label drugs, etc.)

As described previously in our clinical research reports [29], the investigator shall report to the NCGM CRB, MHLW, and the Pharmaceuticals and Medical Devices Agency (PMDA), according to the Clinical Trial Act in Japan. As shown in Table 5, any ① unforeseen death or ② unforeseen diseases that may lead to death are reported to the NCGM CRB, MHLW, and PMDA within 7 days of the recognition of the occurrences after reporting, to the hospital center director of the NCGM and the NCGM Clinical Research Safety Management Office. Foreseen diseases should be reported to the NCGM CRB within 15 days after reporting to the hospital center director of the NCGM and the NCGM Clinical Research Safety Management Office. In addition, the following ③–⑦ diseases that cannot be predicted are reported to the NCGM CRB, MHLW, and PMDA within 15 days after reporting to the hospital center director of the NCGM and the NCGM Clinical Research Safety Management Office.

③ Diseases requiring inpatient hospitalization or the prolongation of existing hospitalization for treatment;

④ Disorders;

⑤ Diseases that may lead to disability, etc;

⑥ From ③ to ⑤, and serious diseases that may result in death or diseases that could be lethal, etc;

⑦ Any congenital diseases or anomalies in the offspring of the treated subject.

Disease reports to the MHLW will be prepared from the disease reports of the jRCT (Clinical Research Protocol and Research Outline Disclosure System).

Diseases suspected to be caused by this study (excluding diseases presented in the above reports) will be reported at the time of periodic reports by the NCGM CRB.

(3) Severity classification of adverse events

The investigator or subinvestigator determines and records the severity of each adverse event. The severity judgment will be determined using the guidance issued by the U.S. Food and Drug Administration [30], and recorded using grades 0–4. Nonapplicable adverse events to the guidance are categorized according to their effects on daily activities as grade 1 (those that do not interfere with daily living activities), grade 2 (those that interfere with daily living activities), grade 3 (those that hinder performing daily living activities), and grade 4 (potentially life-threatening).

### 2.8. The Handling of Cases and Data

The handling of cases and data was performed as per the investigator’s decision, as described in previous clinical studies [29,31].

### 2.9. Quality Control and Assurance

(1) Viewing source documents, monitoring, and auditing

These are performed as described in previous clinical studies that require similar quality standards [29,31].

(2) Efficacy/safety evaluation committee
  The efficacy and safety evaluation committee will hold the following meetings to assess or judge the adverse events or diseases:
  ① In the event of an unexpected serious adverse event related to the test vaccine,
  ② When ≥10 predictable serious adverse events are considered as being related to the test vaccine,
  ③ When the NCGM CRB states its opinions on the continuation of the research,
  ④ When the principal investigator convenes to investigate measures to prevent recurrence,
  ⑤ When consultation is required regarding the judgment and evaluation of the adverse events,
  ⑥ Other convenings by the investigator.

### 2.10. Ethical Considerations

(1) Compliance with laws and regulations

This study will be conducted in compliance with the ethical principles stipulated in the Declaration of Helsinki, the Clinical Research Act, related notifications, and the Act on the Protection of Personal Information (promulgated on 30 May 2003, Law No. 57). The handling procedures, including anonymization and management, storage, and the disposal of samples and information, methods of explanation, and obtaining informed consent from the clinical research subjects, will be performed as described in a previous study [29,31].

(2) Benefits and burdens of the clinical research subjects, anticipated disadvantages in such clinical research, and measures to minimize such burdens and disadvantages

Participating in this study allows for the smallpox vaccination and the prevention of crises or serious forms of the disease when the subject is exposed to the smallpox virus or the MPXV. Moreover, the method that effectively prevents the spread of the infection more efficiently could be investigated by determining the immunogenicity of the test vaccine for the MPXV. As a disadvantage, the following side reactions to the smallpox vaccine may occur:

① adverse reactions

In addition to local inoculation, systemic reactions such as fever, rash, axillary lymph node swelling, malaise, pruritus, urticaria, headache, pain due to myalgia, self-inoculation (a varicella infection caused by the hand inoculation of a virus from the inoculation site to another site), parapox (a vesicle or abscess in the vicinity of the inoculation site), and vaccination eczema (allergic eczema that appears in various forms, such as urticaria and erythema, seen 7–10 days postvaccination) may occur around 10 days postvaccination.

② Clinically relevant findings from nonclinical and other clinical studies

The smallpox vaccine was administered to 268 adults in the 2005 fiscal year in Japan. The perception rate was 91.0% (94.4% for the first-time recipients and 81.7% for secondary recipients), the mean size of redness was 23.8 mm (98 tests), and the mean blister size was 7.6 mm (87 tests). The following adverse events were observed: lymph node swelling, 19.4%; injection site erythema, 5.2%; fever, 1.5%; malaise, 0.7%; postinfection complications (satellite), 0.7%; rash, 0.4%; and injection site swelling, 0.4%; 0.4% self-infection (suspected heterogeneous) was observed [26]. Regarding immunity, neutralizing antibody titers were 4^2.6^ before vaccination (number of tests, 68), 4^5.2^ 1 month postvaccination (number of tests, 39), 4^3.8^ prevaccination (number of tests, 30), and 4^4.8^ 1 month postvaccination (number of tests, 12) in the re-vaccinated subjects, indicating significant antibody titer increments. No adverse reactions were observed for cardiac diseases (chest X-ray and electrocardiogram), encephalitis, parapox, or vaccinia, which were conducted as priority survey items [26].

### 2.11. Payments and Monetary Compensation Related to the Conduct of the Clinical Study

The smallpox vaccine used in this study is transferred from the MHLW, so there is no cost burden on the research subjects. To reduce the burden, we will ensure a ¥3000 cost reduction per hospital visit (including visits for the adverse event consultation and blood sampling).

When a health hazard occurs during the study, the health insurance will be used for the treatment of the subject involved, and the out-of-pocket payments will, in principle, be made by the subjects. The content of insurance coverage and compensation other than insurance coverage was set similarly to previous clinical studies [29].

### 2.12. Conflicts of Interest Management

The management will be performed as described previously [29].

Investigators at the NCGM will submit the NCGM Conflict of Interest Management Committee’s Notification to the NCGM CRB, and continuously check for conflicts of interest throughout the study period for appropriate management and publication. Investigators at the NCGM and NIID have no conflicts of interest.

## 3. Discussion

The smallpox vaccine has been classified into three generations by the WHO. The main first-generation vaccines are Lister strain vaccines used in Europe, and NYBH strain (Dryvax) vaccines prevalent in the United States. These vaccines have strong side effects, especially postvaccinal encephalitis due to neurological complications [32]. ACAM2000 is a second-generation vaccine manufactured by Emergent BioSolutions Inc., and produced by plaque-cloned viruses from Dryvax. The third-generation vaccine is produced using the cell culture of an attenuated virus strain, e.g., the LC168 m-strain vaccine (LC16) and MVA-BN. LC16 has been reported to be superior to conventional smallpox vaccines in efficacy and safety; thus, it has the potential to be an emergency stockpile vaccine in the future [33]. The WHO provisional guidance, issued on 14 June 2022, recommended the use of a third-generation smallpox vaccine, LC16 or MVA-BN [21]. MVA-BN was approved in 2019 in the United States for the “Prevention of mpox in Adults Aged 18 Years and Older with High-Risk Exposure”, based on the results of neutralizing titers against the vaccinia virus in nonclinical and clinical research. It was also approved in Canada in 2020 for the “Prevention of mpox and Related Orthopoxviral Infections” based on the results of studies similar to those conducted in the United States [21]. LC16, as described above, has shown a good response in both first-vaccinated and re-vaccinated individuals [27], and it is the only known vaccine that can be administered to children [25]. Therefore, LC16 has been selected as the preferred vaccine based on the benefit–risk balance when required for those at risk of infection, including children. Although we suppose that this study has the potential to provide important evidence on the prevention of the infection, onset and the aggravation of mpox safely and efficiently, several issues and limitations remain to be considered and addressed in the future.

The primary objective of the present study is to evaluate the immunogenicity of the LC16 against the MPXV by measuring neutralizing antibody titers 28 days after vaccination. In addition, the investigation of the immunogenicity up to 168 days after vaccination will provide key information on the duration of vaccination-induced immunogenicity. Although our results will be limited to just 168 days, they will be regarded as evidence for the efficacy of the anti-mpox vaccination. Based on the results of this study, we are considering the possibility of conducting a larger-scale validation study in the future.

The number of punctures at the time of vaccination used in this study was 5 for the first vaccine and 10 for subsequent vaccines. Previous clinical research conducted in healthy adults with a puncture number of 15 at the LC16 vaccination in the United States showed that the response to neutralizing antibodies to the vaccinia virus and the “take” were similar to those in the study conducted in Japan, with a puncture number of five during vaccination [20,27]. In addition, the safety of the vaccine has been confirmed, with no serious adverse events such as encephalopathy, skin complications, or pericarditis [20]. The multiple-puncture technique applied in the LC16 vaccination has not been required for the accurate collection of the specified liquid quantity. To assess the efficacy and the safety of the LC16 vaccination, an appropriate puncture frequency and inoculum dose may be required.

Healthcare workers who are likely to be exposed to MPO should be vaccinated, according to the WHO [21]. This study was designed as a single-arm study of only 50 healthcare workers in the NCGM; thus, the safety information available in this study will be limited. For instance, pregnant or possibly pregnant individuals were excluded from the study. There is no information on the effects of LC16 administration on women of childbearing potential, and the safety of LC16 in subsequent pregnancies and suckling infants has not been established yet. In clinical trials conducted in Japan so far, more than 97% of the subjects have been male [26,27]. In the clinical study conducted on more than 3000 healthy adults [27], a sample that is more than 60 times the size of that used in this study, two patients experienced adverse events suspected to be severe. Although this study was designed to efficiently verify the immunogenicity conferred by the mpox vaccine against a global epidemic in terms of preventing the spread of the infection over a short period, further large-scale studies need to be conducted in order to comprehensively evaluate the safety and efficacy of the mpox vaccine.

## Figures and Tables

**Table 1:** Inclusion/exclusion criteria and rationales for their setting among the subjects of the clinical trial.

	Items	Rationale for Setting
Inclusion criteria	Individuals who have provided written consent to the principal or agent regarding participation in the study	From an ethical point of view
	Men and women aged ≥20 years at the time of providing informed consent	
	Personnel (physicians, nurses, office workers, assistants, etc.) working at the NCGM who are assumed to be involved in the medical care of patients in the event of an mpox case	From a feasibility and ethical point of view
	Individuals who do not develop smallpox or mpox	Mpox and smallpox are the target diseases to be prevented in this study
Exclusion criteria	Individuals with diseases causing abnormalities in immune function	To ensure the safety of the research subjects by referring to the descriptions in the package inserts
	Individuals undergoing immunosuppressive treatment with corticosteroids, immunosuppressants (cyclosporine, tacrolimus, and azathioprine), and biological agents	
	Individuals with a history of anaphylaxis due to components of the smallpox vaccine	
	Individuals suffering from a fever	
	Individuals with serious acute diseases	
	Women who are or may be pregnant	
	Women who are unable to affirm contraception for four weeks postvaccination	
	The presence of diffuse dermatoses in people who are at risk of disability due to vaccination	
	Individuals with the following risk factors for vaccination, as listed in (i) and (ii) (a)	
	Individuals deemed by the investigator as inappropriate for study inclusion	To ensure the safety of the subjects, the risk should be minimized
	Individuals who were unable to attend the planned research visit at the time of enrollment	To minimize missing data, maintain test accuracy and reduce bias
	Individuals who received a live vaccine within one month of the scheduled test vaccine administration	To ensure the safety of the research subjects as a provision of the general vaccination
	Individuals scheduled to be vaccinated within one month of the scheduled date of the test vaccine administration	Vaccination other than the test vaccine is prohibited for 1 month from the date of administration of the test vaccine

(a) (i) Individuals with a history of a neurological disease such as encephalitis. (ii) Individuals, with or without symptoms, diagnosed by a physician as having the following cardiac or cardiovascular risk factors: 1. Coronary vascular disease; 2. History of myocardial infarction; 3. Angina pectoris; 4. Congestive heart failure; 5. Cardiomyopathy; 6. Transient ischemic attack (symptoms of chest pain but no cardiac damage); 7. Chest pain on exertion, dyspnea, or other heart diseases under the supervision of a physician; 8. Those with three or more of the following five risk factors: Hypertension, hypercholesterolemia, diabetes mellitus, or hyperglycemia; individuals <50 years of age with a relative (parents or siblings) having heart diseases; current smoker.

**Table 2:** Study calendar.

		Obtaining Consent	Inoculation	Observation Period Postvaccination						Out of Appointment	Time of Discontinuation
	Day		0	1–13	14	15–27	28	29–167	168		
	Allowable width				+4 days		+4 days		±7 days		
	Visit		1		2		3		4	Out of appointment	Time of discontinuation
Confirmation before vaccination matters	Qualification	X									
	Obtaining consent	X									
	Subject enrollment	X									
	Confirmation of subjects’ background	X									
	To review concomitant medications	X									
	Vaccination		X								
Blood collection	Evaluation of their immunogenicity		X		X(e)		X(e)		X(e)		
Subject evaluation (subject diary)	Body temperature		X	X	X					X(d)	X(d)
	Chills		X	X	X					X(d)	X(d)
	Headache		X	X	X					X(d)	X(d)
	Fatigue and malaise)		X	X	X					X(d)	X(d)
	Arthralgia		X	X	X					X(d)	X(d)
	Myalgia		X	X	X					X(d)	X(d)
	Skin rash		X	X	X					X(d)	X(d)
	Vomiting		X	X	X					X(d)	X(d)
	Diarrhea		X	X	X					X(d)	X(d)
	Lymphadenopathy		X	X	X					X(d)	X(d)
	Injection site pain		X	X	X					X(d)	X(d)
	Injection site redness		X	X	X					X(d)	X(d)
	Injection site swelling		X	X	X					X(d)	X(d)
	Injection site induration		X	X	X					X(d)	X(d)
Physician Evaluation	Examination		X		X		X		X	X(a)	X(a)
	Local skin reaction on injection site (“take”)				X					X(b)	X(b)
Adverse Event (c)			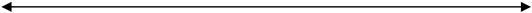								
Diseases affecting immunity and concomitant medications			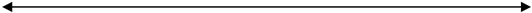								
History of contact with mpox and smallpox subjects and the onset of mpox			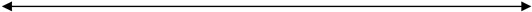								

(a) Up to visit 3, (b) 10–15 days postvaccination, (c) all adverse events until visit 3, and thereafter, only the serious adverse events, (d) Up to visit 2, (e) blood sampling at visits 2, 3, and 4 within an acceptable time period, with the exception of days 14, 28, and 168, should be performed only when feasible. The required items in the designated day and condition are indicated with X.

**Table 3:** Primary endpoint.

Item	Neutralizing Antibody Titer in Visit 1 (a)	Titer Judged as Seroconversion (a)
Neutralizing seroconversion rates against MPXV after smallpox vaccination on day 28, postvaccination	<4	≥16
	≥4	8 times or more than visit 1 titer (b)
	≥1024	undeterminable

(a) The lower and upper limits of the neutralizing antibody titer are set at 2 and 4096, respectively. (b) E.g., if the neutralizing antibody titer is 8 during visit 1, a titer of 64 or more is considered as seroconverted.

**Table 4:** Secondary and exploratory endpoints.

Endpoint	Items	Details
Secondary endpoints	Immunogenicity (a)	Neutralizing seroconversion rates against MPXVs on days 14 and 168
		Neutralizing seroconversion rates on days 14, 28, and 168 against the vaccinia virus
		Rate of the “take”
	Efficacy	Mpox disease until day 168 (b)
	Safety	①All adverse events occurring from postvaccination to visit 3(day 28), ② Deaths due to the adverse events; serious adverse events other than deaths occurring from postvaccination to visit 4 (day 168), ③ Severe (grade ≥ 3) adverse events occurring from postvaccination to visit 4 (day 168).
		Maximum body temperature from postvaccination to 14 days postvaccination
		Physician-reported outcomes (c) ① Specific local adverse events (d), ② Specific systemic adverse events (e), ③ Nonspecific adverse events (f).
		Subject-reported outcomes (c) ① Specific local adverse events (d), ② Specific systemic adverse events (e), ③ Nonspecific adverse events (f).
Exploratory endpoint	Immunogenicity (a)	Neutralizing antibody titers against the MPXV on days 14, 28, and 168 postvaccination
		Neutralizing antibody titers against the vaccinia virus on days 14, 28, and 168 postvaccination

(a) Rationale of immunogenicity: The smallpox vaccine induces neutralizing antibodies against the vaccinia virus in the vaccine, which is important for determining the vaccine’s efficacy. The smallpox vaccine appears to be effective against the MPXV, because the neutralizing antibodies for the vaccinia virus induced by the smallpox vaccine cross to the MPXV [23]. Moreover, in this study, neutralizing antibodies against the vaccinia virus should be measured simultaneously with neutralizing antibodies against the MPXV, to confirm the scientific rationale for the mechanism of action. (b) The onset of mpox should be diagnosed based on the notification criteria stipulated in Article 12, paragraph (1), and Article 14, paragraph (2), of the Act on the Prevention of Infectious Diseases and Medical Care for Patients with Infectious Diseases [28]. (c) Information will be collected via a subject diary and through in-person interviews. (d) Specific local adverse events: injection site redness, swelling, induration, and pain occurring between immediate postvaccination and 14 days postvaccination. (e) Specific systemic adverse events: fever occurring between immediate postvaccination and 14 days postvaccination (fever ≥ 37.5 °C), headache, malaise, vomiting, diarrhea, myalgia, arthralgia, rash, and lymphadenopathy. (f) Nonspecific adverse events: nonspecific adverse events occurring between immediate postvaccination and 14 days postvaccination.

**Table 5:** Reporting of diseases suspected to be caused by the clinical research to the NCGM CRB based on the Clinical Trial Act in Japan.

Research Category	Predictability	Seriousness of Disease	Reporting Deadline
Clinical research using unapproved or off-labeldrugs (a)	Not possible (b)	Death	7 days
		Diseases that may lead to death	
	Possible	Death	15 days
		Diseases that may lead to death	
	Not possible (b)	Diseases requiring inpatient hospitalization or prolongation of existing hospitalization for treatment	15 days
		Disorders	
		Diseases that may lead to disability, etc.	
		Serious diseases in accordance with the above, and diseases that may lead to death	
		Any congenital diseases or anomalies in the offspring of a treated subject	
Diseases suspected to be caused by the clinical research (other than those reported above)			Periodic report

(a) This category was applied when this protocol was developed, i.e., when LC16 was not approved for monkeypox. After approval of LC16 for monkeypox, the disease will be reported according to the research category for the approved drugs. (b) Diseases with not-possible predictability in Table 5 should be reported to the MHLW and the PMDA, as well as to the NCGM CRB.

## Data Availability

Data sharing is not applicable in this article, as no datasets are generated or analyzed in the current study. The protocol for this study is available from the corresponding author upon reasonable request.
